# Identifying the High-Risk Population for COVID-19 Transmission in Hong Kong Leveraging Explainable Machine Learning

**DOI:** 10.3390/healthcare10091624

**Published:** 2022-08-25

**Authors:** Zhihan Jiang, Ka-Man Yip, Xinchen Zhang, Jing Deng, Wilfred Wong, Hung-Kwan So, Edith C. H. Ngai

**Affiliations:** 1 Department of Electrical and Electronic Engineering, Faculty of Engineering, The University of Hong Kong, Hong Kong 999077, China; 2 Department of Paediatrics and Adolescent Medicine, LKS Faculty of Medicine, The University of Hong Kong, Hong Kong 999077, China

**Keywords:** COVID-19, high-risk population, population features, tertiary planning unit, explainable machine learning, SHAP

## Abstract

The worldwide spread of COVID-19 has caused significant damage to people’s health and economics. Many works have leveraged machine learning models to facilitate the control and treatment of COVID-19. However, most of them focus on clinical medicine and few on understanding the spatial dynamics of the high-risk population for transmission of COVID-19 in real-world settings. This study aims to investigate the association between population features and COVID-19 transmission risk in Hong Kong, which can help guide the allocation of medical resources and the implementation of preventative measures to control the spread of the pandemic. First, we built machine learning models to predict the number of COVID-19 cases based on the population features of different tertiary planning units (TPUs). Then, we analyzed the distribution of cases and the prediction results to find specific characteristics of TPUs leading to large-scale outbreaks of COVID-19. We further evaluated the importance and influence of various population features on the prediction results using SHAP values to identify indicators for high-risk populations for COVID-19 transmission. The evaluation of COVID-19 cases and the TPU dataset in Hong Kong shows the effectiveness of the proposed methods. The top three most important indicators are identified as people in accommodation and food services, low income, and high population density.

## 1. Introduction

The World Health Organization has declared coronavirus infectious disease 2019 (COVID-19) a pandemic since 11 March 2020 [1]. As a result of the epidemic, hospitals are overburdened and face significant issues in terms of people staffing, personal protective equipment availability, and intensive care unit bed allocation during the fifth wave in Hong Kong [2]. In this case, slowing down or even cutting off the spread of COVID-19 is vital to relieve the heavy burden on medical systems [3]. As all countries worldwide strive to prevent the growing threat of coronavirus, data on populations, their relative traits, livelihoods, and geography have proven to be invaluable for response efforts to a disease with substantial unknowns [4,5].

Moreover, Hong Kong began administering the fourth round of COVID-19 vaccines to residents aged 18 to 59 in April 2022, with some health experts proposing that the fourth dose be given only to specific groups. The COVID-19 vaccines are unquestionably effective at alleviating the disease, particularly severe symptoms [6]. However, the spatial dynamics of high-risk populations for COVID-19 transmission, which can be helpful for health experts in guiding response efforts such as emergency funding allocation and preventative measures, remain to be further studied.

The development of machine learning algorithms makes it feasible to learn from the available data for accessing the evolving risk factors and the newly exposed areas. Several studies have used supervised machine learning algorithms to identify patients at risk of developing severe COVID-19 symptoms [7]. For example, Assaf et al. [8] predicted risk for critical COVID-19 based on status at admission using machine learning models. Yan et al. [9] developed a machine learning-based model to predict the survival of severe COVID-19 patients with clinical features. In [10], researchers built a machine learning-based model to estimate patients’ health conditions and mortality risk with COVID-19. The majority of them, however, focused on the association between machine learning algorithms and clinical medicine [11], lacking the understanding of spatial dynamics of the high-risk population for COVID-19 transmission. Hyper-local knowledge of what may put people at risk and where to find vulnerable groups can help plan preventative measures and facilitate medical resource allocation, significantly improving the pandemic response to a disease that affects people on a global scale. Therefore, this study aims to investigate the association between population features and COVID-19 transmission risk in Hong Kong.

Moreover, although many state-of-the-art machine learning models achieve remarkable performance in various domains, they are difficult to interpret, hindering their application, especially in healthcare [12]. Fortunately, the recent development of explainable machine learning has enabled the explanation of many complex machine learning algorithms. By leveraging various methods such as feature importance scores, counterfactual explanations, and influential training data, explainable machine learning provides insights into model behaviors [13]. For example, in [14], an explainable machine learning pipeline was proposed to predict material properties. Han et al. proposed a novel degree of locality preservation approach to enhance the explainability of manifold learning [15]. SHAP (SHapley Additive exPlanation) [16] is a game-theoretic approach measuring the importance of features and interpreting the output of machine learning models, which is powerful and has been utilized to explain various techniques and applied to different domains (e.g., healthcare, Internet of Things, and transportation) [17]. For example, Wang et al. proposed an explainable machine learning framework based on SHAP for intrusion detection systems [18]. Ng et al. assessed the mortality and recurrence risk factors of clostridioides difficile infection patients using an explainable machine learning prediction system based on SHAP [19]. SHAP quantifies the contribution of each player (feature) in a collaborative game (the machine learning model) [16]. Unlike many traditional importance analysis methods, the SHAP values can explain the model output globally and locally [20], not only reflecting the importance rank of features in the prediction for each sample but also presenting the different effects of different features on the prediction results in a quantitative way [21].

Therefore, in this work, we first obtained the number of COVID-19 cases and population features for each tertiary planning unit (TPU) (a TPU is a geographic reference system demarcated by the Planning Department for the Territory of Hong Kong, identifying the boundaries of living areas of all individuals [22], detailed in Section 2). Second, we built regression models based on machine learning algorithms to predict the number of cases using population features. Third, we used SHAP values to explain the model output and analyzed the importance and influence of different population features. In this way, the COVID-19 transmission risk of different TPUs can be estimated, and the indicators for high-risk populations can be identified.

We organized our paper as follows. First, we introduce the background, motivation, and objectives of this study in Section 1. Then, we describe the datasets and methodology in Section 2 and report the experiment results in Section 3. Next, we discuss the key findings, implications, limitations, and future work in Section 4. Finally, we conclude this study in Section 5.

## 2. Materials and Methods

In this section, we first describe the datasets we used in this study and the data processing procedure. Then, we introduce the machine learning algorithms used for predicting the number of COVID-19 cases based on population features and the feature importance analysis.

### 2.1. Dataset Description and Processing

The datasets used in this study include the COVID-19 case dataset and the TPU dataset. The data source of COVID-19 is a surveillance system titled “Together, we fight the virus!” designed by the Department of Health of Hong Kong SAR to capture the COVID-19-related vaccination history and provide an update on the infection situation for all residents [23]. The tertiary planning unit (TPU) is a geographic reference system demarcated by the Planning Department for the Territory of Hong Kong [22]. It identifies the boundaries of living areas of all individuals in Hong Kong. Under this boundary system, the whole land area of Hong Kong is divided into 291 TPUs according to the 2016 Population By-census. Each TPU has its socioeconomic features, including demographic, educational, economic, household, and housing variables, characterizing different dimensions of the respective area. Therefore, we explored the relationships between these population features and COVID-19 transmission risk. In this way, high-risk populations for COVID-19 transmission can be timely identified, and corresponding strategies can be implemented to prevent the pandemic outbreak. The study did not require ethical approval because all existing data were retrieved retrospectively and anonymized.

The COVID-19 case dataset contains 10,603 COVID-19 cases in Hong Kong SAR from January 2020 to January 2022, including the age, sex, residential address, and travel history of the cases. There are 5518 females and 5085 males. Their ages range from 12 days to 100 years old (mean (M) = 44.54, standard deviation (SD) = 19.99, in years). We further divided the cases into imported cases, local cases, and unknown cases according to their residential addresses and travel history. It resulted in 1297 imported cases, 9274 local cases, and 32 unknown cases.

The TPU dataset includes the population features in each TPU. Each TPU is identified by a unique three-digit number. As shown in Table 1, these features describe the demographic, economic, industry, housing type, and place of work of the population in TPUs. The total population of TPUs ranges from 1032 to 286,232 (M = 35,784, SD = 41,490). We also calculated the population density for each TPU according to their total population and areas. The household per room is obtained by dividing the number of households by the number of rooms (excluding kitchens, toilets, and bathrooms). Besides the absolute values of different types of populations, we also calculated the proportions of the total population.

Then, we mapped the COVID-19 cases to TPUs according to the residence of cases and finally had 215 TPUs with COVID-19 cases. The number of cases in each TPU ranges from 1 to 708 (M = 49.32, SD = 75.92).

### 2.2. COVID-19 Case Prediction and Feature Importance Analysis

**Figure 1:**
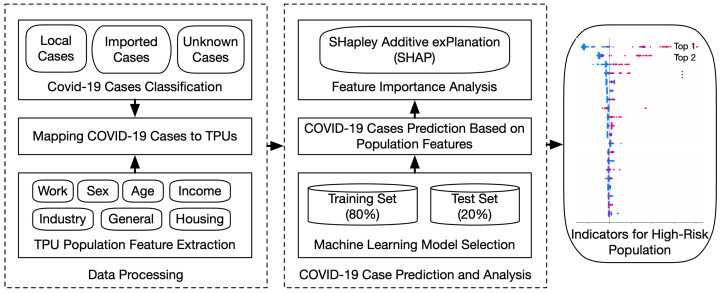
The pipeline of identifying indicators for high-risk COVID-19 transmission population.

To investigate the association between population features and COVID-19 transmission risk, we built the regression model to bridge the population features and the number of COVID-19 cases in TPUs. Then we analyzed the importance and influence of features on the prediction results to identify important population features. The pipeline is shown in Figure 1.

We divided the 215 TPUs into a training set (172 TPUs, 80%) and a test set (43 TPUs, 20%), and trained regression models on the training set using the following algorithms.

- *Linear Regression (LR)* is a linear approach for modeling the relationship between features and output [24]. The objective is to minimize the residual sum of squares between the ground-truth values and the predicted values by the linear approximation. It is low-cost and easy to implement.
- *K-Nearest Neighbor Regression (KN)* only consider the k-nearest data samples assuming that the predicted value should be in the neighborhood. It averages the observations in the same neighborhood to model the relationship between features and output [25]. It requires few parameters, is easy to implement, and can be applied to various linear or non-linear regression tasks.
- *Decision Tree (DT)* is a model with a tree structure including the root node, internal nodes, leaf nodes, and branches, aiming to make a rational decision based on the features of the training set by answering all the questions on root and internal nodes [26]. The leaf nodes can be categories or real numbers, making it applicable to both classification and regression problems. Since every decision is made by all the information on each layer of the tree, it has good interpretability and is easy to understand.
- *Random Forest (RF)* consists of several randomly created decision trees, and the output prediction is aggregated from predictions of these decision trees, i.e., the average of outputs [27]. A random forest regression model is trained by constructing multiple decision trees with the training dataset in parallel. When testing the model, a new data point will go through all the decision trees, and the result will be the average value across all the predicted values. It can achieve high accuracy and is a robust algorithm.
- *XGBoost (XGB)* is eXtreme Gradient Boosting, an efficient implementation of distributed gradient boosting that can be used for building regression model [28]. It provides parallel tree boosting and has achieved outstanding performance in various domains. It stands out from other algorithms due to its high efficiency, low computational cost, good performance, and generalization [29].

The above-mentioned algorithms have been applied to various domains and achieved remarkable performance. Compared with many other machine learning and deep learning algorithms, they are easier to implement, more efficient, explainable, and require less data for training. We trained models based on these algorithms and selected the one with the best performance for further analysis.

For each model, the parameters are determined by grid search and validated through five-fold cross validation [30] on the training set. Then, we compared the performance of the models on the test set and select the one with the best performance for further analysis. Specifically, for LR and KN, we used principal components analysis (PCA) to reduce the influence of multicollinearity [31]. The explained variance in PCA is set to 95%. We used the $R^{2}$ score as the coefficient of determination, which is commonly used to evaluate the performance of a regression model. It depends on the ratio of total deviation of results described by the model. A higher $R^{2}$ score value indicates a higher proportion of data points within the line created by the regression equation. Therefore, a higher $R^{2}$ score means better performance. The formula for the $R^{2}$ score is given as follows:

(1) $$R^{2}=1-S_{r}/S_{t}.$$

Here, $S_{r}$ is the sum of squares of the residual errors and $S_{t}$ is the total sum of the errors.

We also report the mean absolute error (*MAE*) of the prediction results. *MAE* measures the errors between the predicted and observed values. It is calculated as the sum of the absolute errors divided by the sample size, detailed as follows:

(2) $$MAE=\frac{\sum_{i=1}^{n}\left|y_{i}^{predicted}-y_{i}^{groundtruth}\right|}{n},$$

where $y_{i}^{predicted}$, $y_{i}^{groundtruth}$, and *n* are the predicted value, ground truth value, and the size of the test set.

After selecting the model with the best performance, we conducted the feature importance analysis on the regression model leveraging SHAP [16] to rank the importance of different population features and analyze the influence of important features on the prediction results. More specifically, suppose $X=\{\left(x_{i},y_{i}\right)|i=1,2,\ldots ,N\}$ is the training dataset, where $x_{i}$ and $y_{i}$ are the input and output of *i*th sample, and *N* is the number of samples. We denote the *j*th feature of $x_{i}$ by $x_{ij}$, and the number of features is *M*. Then, the SHAP values follow the equation below:

(3) $$y_{i}=E\left[f\left(X\right)\right]+\sum_{j=1}^{M}f\left(x_{ij}\right),i=1,2,\ldots ,N,$$

where E[f(X)] is the baseline value of the whole model (the expected output, which is usually the mean of the predicted value of all samples), and $f(x_{ij})$ is the SHAP value of $x_{ij}$, denoting the contribution of the *j*th feature in the prediction of *i*th sample to $y_{i}$. The SHAP values of features reflect their impacts on the model output. A positive SHAP value ($f(x_{ij})>0$) increases the number of COVID-19 cases predicted, while a negative SHAP value decreases the output. The mean values of the absolute SHAP values for features are used to rank the importance of features [32]. By analyzing the SHAP values and importance rank of features, we can figure out the influence of different features on the prediction results so as to identify indicators for COVID-19 transmission risk in TPUs and select the most important features required to achieve good performance.

Furthermore, we normalized the SHAP values of features with the following equation:

(4) $$\tilde{SHAP(i)}=\frac{SHAP(i)}{\sum_{j=1}^{N}SHAP\left(j\right)},$$

where $\tilde{SHAP(i)}$ and SHAP(i) are the normalized SHAP value and raw SHAP value of feature *i*, and *N* is the number of original features (N = 74). Therefore, $\sum_{j=1}^{N}SHAP\left(j\right)$ is the sum of the SHAP values of the 74 original features, and the normalized SHAP value of feature *i* ($\tilde{SHAP(i)}$) is the proportion of the raw SHAP value of feature *i* in the sum of SHAP values of all features. Then, we selected the top n features when their cumulative normalized SHAP values account for more than 90% of the sum of all normalized SHAP values, i.e., $\sum_{i=1}^{n}\tilde{SHAP(i)}>0.9$, and retrained the models based on the selected important features. In this way, the redundant features can be excluded to reduce the model complexity [19].

## 3. Results

In this section, we elaborate on the results of COVID-19 case prediction and feature importance analysis.

### 3.1. COVID-19 Case Prediction

**Figure 2:**
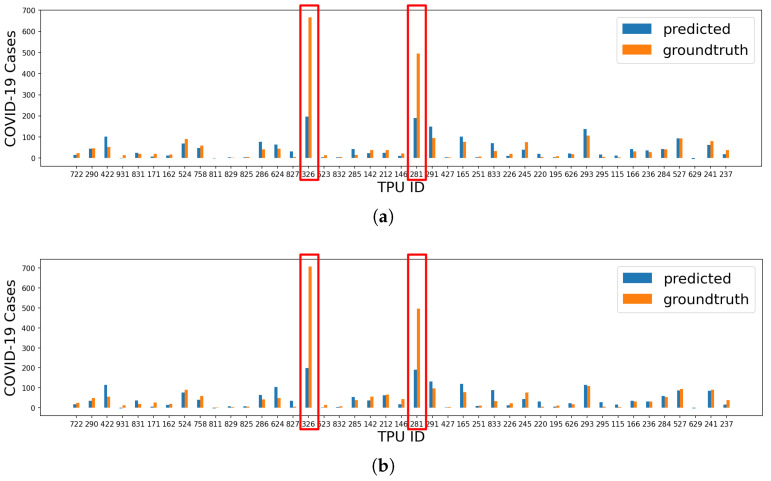
The prediction results of XGB for all TPUs. (**a**) Prediction results of local cases for all TPUs ($R_{0,0}^{2}=0.470$, MAE=31.931). (**b**) Prediction results of all cases for all TPUs ($R_{0,1}^{2}=0.444$, MAE=34.566).

The prediction results of COVID-19 cases are shown in Table 2 ($R_{0,0}^{2}$ and $R_{0,1}^{2}$). We can find that the $R^{2}$ scores of all algorithms are less than 0.5, indicating a disappointing regression performance. However, after looking into the prediction results, we find that the model achieves good performance except for some TPUs with extremely high COVID-19 cases, as shown in Figure 2. We look into the top three TPUs with the highest number of COVID-19 cases. The TPU with the most COVID-19 cases (TPU ID: 326, 667 local cases, 37 imported cases, and 4 unknown cases) is located in Kwai Chung where there was a large-scale outbreak in a few housing estates, including Kwai Chung Estate, Tai Wo Hau Estate, and Kwai Shing West Estate. There were many cases involving residents working at hospitals and elderly homes. Residents also complained that they had to queue for a long time to get tested at mobile testing stations, increasing the cross-contamination risk [33]. The TPU with the second highest cases (TPU ID: 281, 495 local cases, 2 imported cases) is located in Tsz Wan Shan, which belongs to Wong Tai Sin District with a high poverty rate and a large number of public housing estates for lower socioeconomic groups [34]. The TPU with the third highest cases (TPU ID: 225) is located in Yau Ma Tei, which is an older and well-known district attracting many tourists and locals. Based on the above observations, we believe that the environment of the housing estates may play a more significant role than the general regional characteristics of the TPU. Further studies should be conducted on these special cases. Therefore, we removed the top three TPUs with the highest COVID-19 cases and retrain the model. In this case, the performances of KN, RF, and XGB significantly improve, achieving $R^{2}$ scores above 0.8 ($R_{1,0}^{2}$ and $R_{1,1}^{2}$ in Table 2).

**Figure 3:**
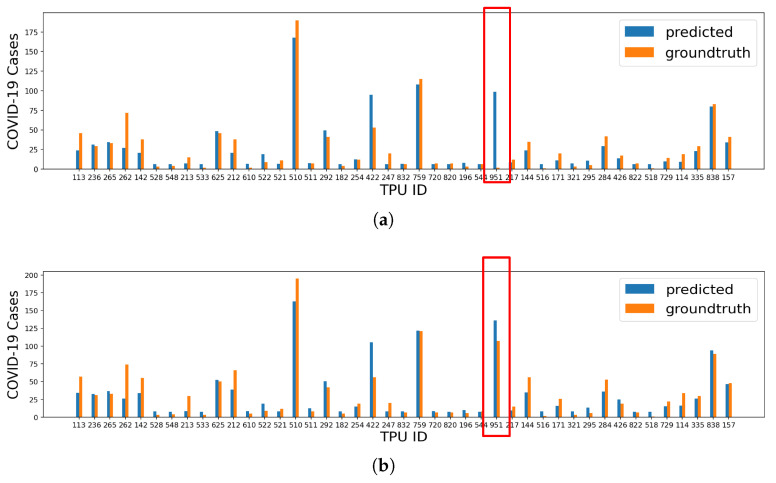
The prediction results of XGB for TPUs excluding the top 3 TPUs. (**a**) Prediction results of local cases for TPUs excluding the top 3 TPUs ($R_{1,0}^{2}=0.695$, MAE=10.136). (**b**) Prediction results of all cases for TPUs excluding the top 3 TPUs ($R_{1,1}^{2}=0.830$, MAE=10.490).

**Figure 4:**
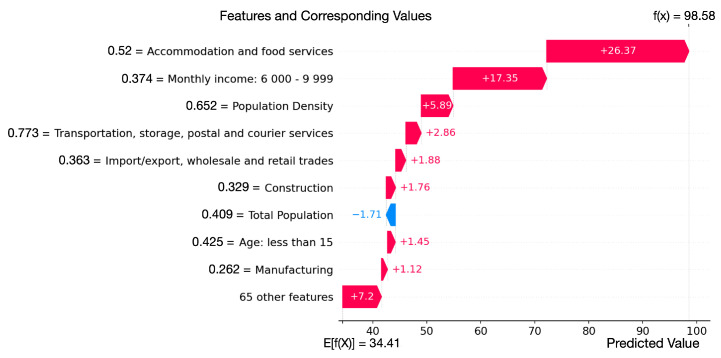
The influence of population features of TPU with ID 951 on prediction.

In general, XGB achieves the best performance with $R^{2}$ scores of 0.695 and 0.830 for local cases and all cases, respectively, as shown in Figure 3. The MAE local case prediction is 34.566, and the MAE on the prediction of all cases is 10.490. Specifically, we find that for the TPU with ID = 951, the model shows high accuracy in terms of total case prediction, while in terms of local case prediction, there is a big gap between the ground-truth value (2 cases) and prediction value (99 cases). This TPU is Chek Lap Kok, which has many hotels and an international airport. Therefore, it has many imported cases (105 cases) and only two local cases. However, as shown in Figure 4, the larger number of hotels increases the population working in accommodation and food services, and thus increases the number of COVID-19 cases predicted. This case shows that the model performs better in total case prediction, and more features about the environment of TPUs are required to predict the number of cases in a more fine-grained manner.

### 3.2. Feature Importance Analysis

**Figure 5:**
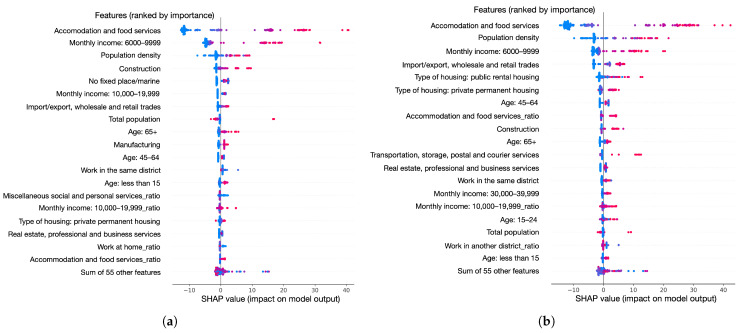
The overview of the features’ SHAP values. The features are ranked by importance (the most important is at the top). Red color means higher feature values and blue means lower feature values. Lower SHAP values mean lower COVID-19 cases. (**a**) Top 20 most important features and the sum of 55 other features for the prediction of local cases. (**b**) Top 20 most important features and the sum of 55 other features for the prediction of all cases.

We explore the importance and influence of population features to find high-risk populations for COVID-19 transmission using SHAP. The overview of the SHAP values of the features for predicting local cases and total cases are shown in Figure 5a,b, respectively.

We can find that the population working in accommodation and food services, the population with a monthly income between 6000 and 9999, and the population density are the top three most important features. For local case prediction as well as all case prediction, the number of cases increases with the increase of the population working in accommodation and food services, population density, and population with income between 6000 and 9999 (a lower income in Hong Kong SAR). Moreover, the local cases increase with the increasing population working in construction, manufacturing, and population with age ≥65. The number of total cases increases with the increase of the population with the population working in import/export, wholesale and retail trades, construction, transportation, storage, postal and courier services, population with private permanent housing, and public rental housing.

Then, we normalized the SHAP values according to Equation (4) and selected the top n features when their cumulative normalized SHAP values account for more than 90% of the sum of all normalized SHAP values. In this way, we selected the top 37, 41, 18, and 16 most important features for local case prediction on all TPUs, total case prediction for all TPUs, local case prediction for TPUs excluding the top three TPUs, and total case prediction for TPUs excluding the top three TPUs, respectively. We retrain the models based on the selected important features, and the results are shown in Table 2 ($R_{0,0,s}^{2}$, $R_{0,1,s}^{2}$, $R_{1,0,s}^{2}$, and $R_{1,1,s}^{2}$). We can find that the models based on the important features can achieve close or even better performance compared with using the original 74 features, which shows the effectiveness of the importance rank and the importance of these features, and the model complexity can be significantly reduced.

## 4. Discussion

This study explored the impacts of population features on the transmission risk of COVID-19. We built machine learning models to predict the number of COVID-19 cases using the population features in TPUs and evaluated the influence of different features by explaining the model using SHAP. The top three most important indicators for high-risk COVID-19 transmission population are identified as people in accommodation and food services, low income, and high population density.

According to our findings, it is interesting that the population working in accommodation and food services plays the most significant role, not healthcare workers [35]. TPUs with more of this type of population are predicted to have more COVID-19 cases. Occupational exposure may be related to different characteristics of their works [36], particularly in the accommodation and food service sub-sectors, where human interaction is essential [37]. There is a lot of interaction between customers and employees in restaurants and hotels. The Designated Quarantine Hotel Scheme has been fully implemented since December 2020 in Hong Kong. Those designated hotels solely receive travelers arriving from foreign places for compulsory quarantine. Therefore, the workers and their representatives in the accommodation and food services sub-sectors are at risk of infection. Their irregular operations at work may bring the virus from the quarantined passengers to local residents. This is especially important today because ensuring workplace safety and health is critical for managing the COVID-19 pandemic, particularly in these sub-sectors. It can also benefit the monitoring and updating of the knowledge available about COVID-19, including prevention of transmission and the management of suspected or confirmed cases. A checklist has been designed as a tool to help implement and continuously improve practical action to prevent and mitigate the spread of COVID-19 in accommodation and food service activities established by the International Labour Organization [37]. Restaurant, hotel, and bar staff should regularly practice hygiene practices (frequent handwashing, respiratory hygiene, and frequent cleaning/disinfection of work surfaces and touch points) in accordance with WHO COVID-19 guidance for food businesses on food safety [38].

Moreover, special attention should be paid to areas with high population density areas and lower-income populations. Co-locating overcrowdedness and residents from unfavored socioeconomic classes usually synergistically increase the vulnerability among them and result in the spread of within-neighborhood transmission [39,40,41]. Therefore, the authority should provide more resources in the related lower-income and high-population districts.

Furthermore, more prevention measures should be taken for the aged care centers where there are many old people who are more likely to have severe symptoms gathering together. Unfortunately, in the fifth wave of COVID-19 in Hong Kong, many aged care centers witnessed the outbreak of the pandemic, causing a lot of deaths. As shown in our analysis, the number of local cases is positively related to the population with age ≥65, which is possibly because the elderly have a lower vaccination rate and are more susceptible to the virus [42]. Another important feature is the population working in construction. This may be due to sharing changing rooms and not wearing masks properly at work because of the working environment [43]. Additionally, the number of cases increases with the increase of the population working in import/export, wholesale and retail trades, transportation, storage, postal and courier services, since they have to touch a lot of domestic and overseas goods, increasing the risk of exposure to the virus. We suggest that those critical groups should be eligible for vaccination with the fourth dose.

### 4.1. Implications

Our study demonstrates that the population features of TPUs can be used to accurately predict the number of COVID-19 cases using machine learning algorithms. Apart from the population features, more fine-grained environmental features of TPUs can be used to improve the prediction performance. Furthermore, by leveraging the explainable machine learning, we can find the influence of different population features on the transmission of COVID-19 and identify indicators for high-risk populations for COVID-19 transmission effectively and efficiently. The analysis provides implications for policymakers to pay more attention to the populations with a high risk of COVID-19 transmission. The predicted COVID-19 cases can be significantly reduced by improving the critical features. The proposed pipeline can help investigate the dynamic feature importance and influence to facilitate the update of preventative strategies adaptively.

It also indicates the power of explainable machine learning, and the pipeline can also be applied to other tasks in public health studies. For example, people’s contextual information, such as demographics and environmental conditions, can be used to predict their health status, and by explaining the prediction model, the influence of different contexts can be analyzed. It can also be applied to classify the risk of pandemic transmission for different areas based on environmental features, and by evaluating the influence of environmental features, policymakers can take corresponding strategies to reduce the risk.

### 4.2. Limitations and Future Work

We discuss the following limitations of our work. First, we find that our model performs well on most TPUs except for some TPUs with specific characteristics where there were large-scale outbreaks of COVID-19, such as areas with many quarantine hotels and aged care centers. To address this issue, besides the population features of TPUs, the environmental features, such as points of interest and built-environment features, can be incorporated to improve the prediction performance. Furthermore, we plan to improve the existing machine learning algorithm to make it more adaptive to the prediction of COVID-19 cases based on environmental features.

Second, it is difficult to directly figure out the non-monotonic association between the population features and the number of COVID-19 cases based on the SHAP values. However, the importance rank of features can help us prune some unimportant features. In this case, we can target those crucial features to investigate their complex influences on the prediction result.

Third, because of the limited data size and considering the interpretability of the model, we use no deep learning-based methods [44]. However, the development of transfer learning and explainable deep learning has made it possible to train a deep learning model with a small dataset and explain it. In the future, we plan to compare the current model with more state-of-the-art deep learning models. Additionally, most of the COVID-19 cases included in this study are from the first to fourth waves of COVID-19 in Hong Kong. However, the COVID-19 cases significantly surge in the fifth wave. In the future, we plan to conduct more analyses after collecting more COVID-19 cases.

## 5. Conclusions

We built machine learning models to predict COVID-19 cases accurately and identify high-risk populations for COVID-19 transmission effectively. Response efforts such as resource allocation and preventative measures can be implemented more efficiently. We believe policymakers should pay more attention to the high-risk groups identified and apply further interventions to reduce risk or prioritize vaccination for them.

## Figures and Tables

**Table 1:** Population features.

Type of Features	Features
General Information	Total population
	Population density
	Household per Room (excluding kitchens and toilets/bathrooms)
Population by Sex	Male
	Female
Population by Age	<15
	[15, 24]
	[25, 44]
	[45, 64]
	≥65
Population by Type of Housing	Public rental housing
	Subsidised homeownership housing
	Private permanent housing
	Non-domestic housing
	Temporary housing
Population by Monthly Income	<6000
from Main Employment	[6000, 9999]
(excluding unpaid family workers, in HKD)	[10,000, 19,999]
	[20,000, 29,999]
	[30,000, 39,999]
	[40,000, 59,999]
	≥60,000
Population by Place of Work	Work in the same district in Hong Kong
	Work in another district in Hong Kong
	No fixed place/marine
	Work from home
	Places outside Hong Kong
Population by Industry	Manufacturing
	Construction
	Import/export, wholesale and retail trades
	Transportation, storage, postal, and courier services
	Accommodation and food services
	Information and communications
	Financing and insurance
	Real estate, professional, and business services
	Public administration, education, human health, and social work activities
	Miscellaneous social and personal services
	Others

**Table 2:** Results of COVID-19 cases prediction.

Results	LR	KN	DT	RF	XGB
$R_{0,0}^{2}$	0.421	0.343	0.491	0.398	0.470
$R_{0,1}^{2}$	0.429	0.333	0.445	0.383	0.444
$R_{1,0}^{2}$	0.047	0.615	0.687	0.631	0.695
$R_{1,1}^{2}$	0.394	0.801	0.248	0.812	0.830
$R_{0,0,s}^{2}$	0.464	0.347	0.512	0.416	0.428
$R_{0,1,s}^{2}$	0.471	0.357	0.382	0.390	0.440
$R_{1,0,s}^{2}$	0.364	0.641	0.456	0.624	0.698
$R_{1,1,s}^{2}$	0.688	0.811	0.321	0.812	0.838

$R_{0,0}^{2}$ and $R_{0,1}^{2}$ are the $R^{2}$ scores of the local case and total case prediction for all TPUs. $R_{1,0}^{2}$ and $R_{1,1}^{2}$ are the $R^{2}$ scores of the local case and total case prediction for TPUs excluding the top 3 TPUs. $R_{0,0,s}^{2}$ and $R_{0,1,s}^{2}$ are the $R^{2}$ scores of the local case and total case prediction with reduced features (the top 20 most important features) for all TPUs. $R_{1,0,s}^{2}$ and $R_{1,1,s}^{2}$ are the $R^{2}$ scores of the local case and total case prediction with reduced features (the top 20 most important features) for TPUs excluding the top three TPUs. In KN, n_neighbors=8. In DT, max_depth=8 and min_samples_split=9. In RF, max_depth=8, min_samples_split=4, and n_estimators=450. In XGB, for training on all TPUs, n_estimators=450, learning_rate=0.06; for training on TPUs excluding the top 3 TPUs, n_estimators=200, learning_rate=0.01; moreover, max_depth=2, min_child_weight=1, subsample=0.8, colsample_bytree=0.8, reg_alpha=0, reg_lambda=0. Other parameters followed the default setting.

## Data Availability

Not applicable.

## Abbreviations

The following abbreviations are used in this manuscript:

COVID-19	Coronavirus Infectious Disease 2019
TPU	Tertiary Planning Unit
SHAP	SHapley Additive exPlanation
LR	Linear Regression
KN	K-Nearest Neighbor Regression
DT	Decision Tree
RF	Random Forest
XGB	eXtreme Gradient Boosting
